# Book Notices

**Published:** 1870-04

**Authors:** 


### Book Notices.

The Cell Doctrine; its History and Present State. For the use of students in medicine and dentistry. Also a copious Bibliography of the subject. By JAMES TYSON, M.D., Lecturer on Microscopy in the University of Pennsylvania, and on Physiology in the Pennsylvania College of Dental Surgery; Fellow of the College of Physicians of Philadelphia, &c. With a colored plate, and other illustrations. Philadelphia: LINDSAY & BLAKISTON. 1870.

This is a small-sized octavo volume, of 150 pages, on excellent paper and type, with good illustrations. It presents a very interesting summary of the history and present status of the doctrines concerning cell development and function. For sale by S. C. Griggs & Co., Chicago. Price $2.00.

---

A Manual of Clinical Medicine and Physical Diagnosis. By THOMAS HAWKS TANNER, M.D., F.L.S., &c. Third American from the Second English Edition. Revised and enlarged by TILBURY FOX, M.D., London, Physician to the Skin Department of the University College Hospital. Philadelphia: HENRY C. LEE. 1870.

This is a neatly published duodecimo volume, of 366 pages. It will be found a very convenient and useful little manual, especially for advanced students and young practitioners.

---

A Practical Treatise on the Diseases of Children. By J. FORSYTH MEIGS, M.D., one of the Physicians to the Pennsylvania Hospital; Consulting Physician to the Children's Hospital; Fellow of the College of Physicians, of Philadelphia, &c.; and WILLIAM PEPPER, M.D., one of the Physicians to the Philadelphia Hospital; Lecturer on Morbid Anatomy, at the University of Pennsylvania; Pathologist to the Pennsylvania Hospital, &c. Fourth Edition of Meigs on Diseases of Children, revised and greatly enlarged. Philadelphia: LINDSAY & BLAKISTON. 1870. This is a full-sized octavo volume, of 921 pages.

We have not had time to examine this very full, and, doubtless, able treatise on the diseases of children; but, from the character of the smaller work of Dr. Meigs, and the well known reputation of both authors, we do not hesitate to recommend it to the profession.

---

DR. DIEULAFOY'S "ASPIRATEUR SOUSCUTANE."—Under this name, an instrument has been suggested, by means of which effusions into synovial or serous membranes, collections of pus or blood, and even hydatid sacs, may be safely evacuated. It consists of an instrument resembling a subcutaneous injection syringe, with a terminal and a lateral tube fitted with stopcocks, to which a *capillary* trocar can be fitted, so that after withdrawal of the morbid liquid, an injection may be practiced without removing either the trocar or the pump.—*Medical Times and Gazette, Nov. 20, 1869.*

